# Interfacial Engineering of Graphene Nanosheets at MgO Particles for Thermal Conductivity Enhancement of Polymer Composites

**DOI:** 10.3390/nano9050798

**Published:** 2019-05-24

**Authors:** Wu Pan, Miaomiao He, Li Zhang, Yi Hou, Chen Chen

**Affiliations:** 1 Analytical and Testing Center, Sichuan University, Chengdu 610065, China; panwupanwu@foxmail.com (W.P.); mmiao_he@163.com (M.H.); zhangli9111@126.com (L.Z.); 18780219802@163.com (Y.H.); 2 Department of Chemical and Biomolecular Engineering, University of California, Los Angeles, CA 90095, USA

**Keywords:** graphene, polymer composites, thermal conductivity, interfacial adhesion

## Abstract

An important task in facilitating the development of thermally conducting graphene/polymer nanocomposites is to suppress the intrinsically strong intersheet π-π stacking of graphene, and thereby to improve the exfoliation and dispersion of graphene in the matrix. Here, a pre-programmed intercalation approach to realize the in situ growth of graphene nanosheets at the inorganic template is demonstrated. Specifically, microsized MgO granules with controlled geometrical size were synthesized using a precipitation method, allowing the simultaneous realization of high surface activity. In the presence of a carbon and nitrogen source, the MgO granules were ready to induce the formation of graphene nanosheets (G@MgO), which allowed for the creation of tenacious linkages between graphene and template. More importantly, the incorporation of G@MgO into polymer composites largely pushed up the thermal conductivity, climbing from 0.39 W/m∙K for pristine polyethylene to 8.64 W/m∙K for polyethylene/G@MgO (60/40). This was accompanied by the simultaneous promotion of mechanical properties (tensile strength of around 30 MPa until 40 wt % addition of G@MgO), in contrast to the noteworthy decline of tensile strength for MgO-filled composites with over 20 wt.% fillers.

## 1. Introduction

In the areas of thermal management, heat dissipation and heat exchanging materials, intrinsic low thermal conductivity (TC) remains the main challenge for the application of polymer composites, regardless of low weight, desirable processing feasibility, good corrosion resistance, and low cost [1,2]. Incorporation of thermally conducting fillers, such as aluminum nitride [3], boron nitride [4], carbon nanotubes [5] and graphene nanosheets [6], represents the most common and affordable approach to TC promotion of polymers. Featuring extremely high TC values in the range of 3080–5150 W/m∙K [7], graphene has garnered intense interest in the scientific and industrial communities. The graphene-enabled TC improvement, property improvement and multifunction are mainly related to the large surface area and high surface activity, which allow the creation of thermally conductive networks with high structural integrity and excellent reinforcing efficiency [8,9,10].

The surface chemistry of graphene is of importance for the stacking order and dispersion morphology in polymer composites [11], which profoundly affect the physicochemical properties of graphene/polymer composites, including the mechanical performance and TC, which are both linked to application-specific configurations [12]. Taking into account the large surface area and interaction activity of graphene, undesirable agglomeration of graphene in the matrix is frequently encountered, ultimately leading to the deterioration of comprehensive properties. Thus the local aggregation in the matrix, to a large extent, probably negates any benefits associated with the nanoscopic merits of graphene [13]. This has generated a demand for sufficient exfoliation and uniform dispersion of graphene in the pursuit of high-performance graphene/polymer composites with improved TC.

Emerging as two main routes to harvest the specialties of graphene in development of polymer composites, surface modification and pre-programmed intercalation of graphene sheets have been used to tailor the dispersion morphology and the interfacial adhesion. The former pathway focuses on the covalent decoration of sheet surfaces with functional groups like small molecules or special chemical structure, which was hypothesized to decrease the intersheet interactions and improve the exfoliation of graphene sheets in the polymer matrix [14,15]. For instance, polyvinyl pyrrolidone was used as a dispersant of graphene nanosheets to improve the dispersion morphology in polyethylene, although displaying limited effect in TC promotion (below 1.2 W/m∙K) [16]. As in the case of pre-programmed intercalation, Hakkarainen et al. proposed the use of medium platform to immobilize the nanofillers, which paved a straightforward route to multiscale morphology control and multiple property improvements [17]. This methodology not only allows for the facile yet effective control on the nanofiller morphology, but also enables the straightforward fabrication of high-performance composites.

While recognizing the promising potential to develop high-performance graphene nanocomposites using the pre-programmed intercalation principles, we attempt the launch of hierarchical structure design by introducing inorganic platform to trigger the formation of graphene nanosheets. In the proposed conception, our key elements are (1) rational design of a reliable template that is highly active to induce the growth of graphene; (2) appropriate regulation of the template size at the considerable level with graphene sheets; (3) strong interfacial bonding to endows the promotion of mechanical properties by incorporation of the well-organized graphene-template entities; (4) facile and affordable generation of graphene at the template that supports the large-scale and low-cost fabrication of graphene nanocomposites. This effort sheds light on the flexibility in the reliable regulation of hierarchically structured nanofillers by the in situ formation of graphene nanosheets at the templates, that encourage the pursuit of high-performance graphene nanocomposites toward an optimized balance of TC gains and mechanical property enhancement.

## 2. Materials and Methods

Sodium carbonate (Na_2_CO_3_), magnesium chloride (MgCl_2_), HCl, distilled water and other chemicals were obtained from Sinopharm Chemical Reagent Co., Ltd. (Shanghai, China), and were used as received.

Using a precipitation method, aqueous Na_2_CO_3_ solution (0.11 mol/mL) was gently added to aqueous MgCl_2_ (0.1 mol/mL), which was accompanied by mechanical mixing. The mixture was sent to hydrothermal reaction in a reactor at 200 °C and 0.6 MPa within 30 min, yielding the formation of MgCO_3_ granules with highly ordered crystal plates [18]. The precipitated MgCO_3_ particles were filtered, followed by drying at 110 °C for 6 h. The MgCO_3_ granules were calcined to MgO granules at 500 °C for 4 h under nitrogen atmosphere.

Using the MgO granules as the template, porous nanocarbons were grown at MgO by the chemical vapor deposition (CVD) method. The MgO granules were heated to 600 °C in a tube furnace at a rate of 20 °C/min and annealed at 600 °C for 0.5 h. The growth of graphene was triggered with the introduction of H_2_ (100 sccm) into the gas bubbler containing acetonitrile, and the CVD reaction was held for 0.5 h to obtain the sufficient formation of graphene layers at the MgO template (G@MgO). After that, nitrogen was used to purge away any remaining H_2_, during the cooling procedure.

Using a HAAKE mill, the G@MgO was melt compounded with high-density polyethylene (PN049, Saudi Basic Industries Corporation, Tianjin, China) at 200 °C with a filler content ranging from 5 wt % to 40 wt %. The composites were compression molded into thin films with a thickness of 2 mm.

An SE-4800 scanning electron microscopy (SEM) (Hitachi, Tokyo, Japan), operating at an accelerated voltage of 5 keV, was employed to trace the morphological features of the MgCO_3_ precursors, the MgO template, the G@MgO particles, and the mesoporous graphene without MgO template, as well as the fracture morphology of polyethylene composites. To observe the mesoporous graphene, the G@MgO particles were immersed in 1 M HCl solution for 24 h to etch the interior MgO template, followed by vacuum drying at 120 °C for 8 h. All the samples were sputter-coated with a 3.5 nm thick gold layer prior to the SEM observations. 

Transmission electron microscopy (TEM) was used to image the morphology of graphene induced by the MgO template. Mesoporous graphene was obtained using the same method for SEM observation. Droplets of G@MgO and mesoporous graphene suspensions in ethanol were deposited onto a lacey carbon film 400 mesh copper TEM grid (Ted Pella, Inc., Redding, CA, USA) and allowed to dry in ambient conditions prior to TEM imaging (Hitachi HT7700, 80 keV).

N_2_ adsorption–desorption isotherms were measured on a Micromeritics ASAP 2020 at 77 K, using vacuum dried samples (150 °C/6 h). The isotherms were analyzed using the two-parameter Brunauer–Emmett–Teller (BET) model for specific surface area, the Dubinin–Radushkevitch model for micropore volume, the Horvath–Kawazoe method for micropore size distribution, and non-local density functional theory (NLDFT) isotherm fitting for meso–macropore size distribution.

Raman spectroscopy was performed on a Thermo Nicolet Almega XR Dispersive Raman microscope, comprising a 0.9 numerical aperture microscope objective, a ×50 lens and 10 exposures of 5 s. The laser wavelength is 532 nm (24 mW).

Following the standard GB/T3399-1982, the guarded hot plate method was used to evaluate the thermal conductivity (TC) of the composites on a DRH-V (Xiangyi Instrument, Xiangtan, China) at 25 °C.

Following the ASTM standard D638, tensile testing was performed on an Instron universal test instrument (Model 5944, Instron Instruments, Norwood, MA, USA) with a load cell of 500 N at 23 °C and relative humidity of 50%. The crosshead speed was set at 50 mm/min and the gauge length was 20 mm.

The composites were machined to thin films (thickness of ~20 μm) and were imaged on an Olympus BX43 microscope equipped with a digital camera. 

## 3. Results and Discussion

**Figure 1:**
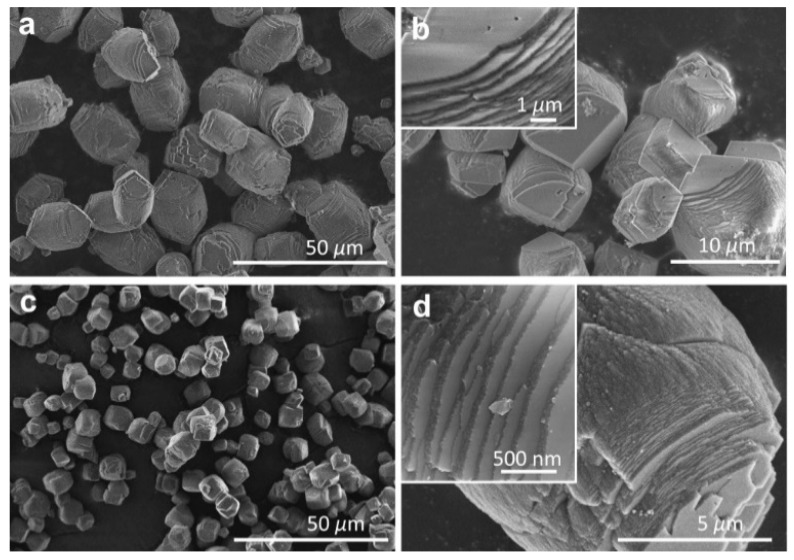
SEM micrographs showing the structural features of (**a**,**b**) MgCO_3_ and (**c**,**d**) MgO assembled by nanosized laminates.

The morphological features of MgCO_3_ precursors prepared by precipitation were directly examined by SEM observations (Figure 1a,b). It is clearly shown that the microsized MgCO_3_ granules were orderly assembled by wafer-like nanosheets, unlike the general cubic structure with epitaxial growth along one crystalline direction [19]. The size of MgCO_3_ granules was well controlled in the range of 5–10 μm, providing desirable platforms for the growth of graphene with microsized planar sheets [20]. Upon high-temperature decomposition, the MgCO_3_ precursors were transformed to MgO templates with slightly decreased size, accompanied by the degradation of sheet thickness (Figure 1c,d). Moreover, a large amount of mesopores were created during the release of CO_2_, affording the enhancement of surface activity. With respect to the geometrical size and surface activity of the hard templates, the growth of graphene could be well controlled by the CVD method.

**Figure 2:**
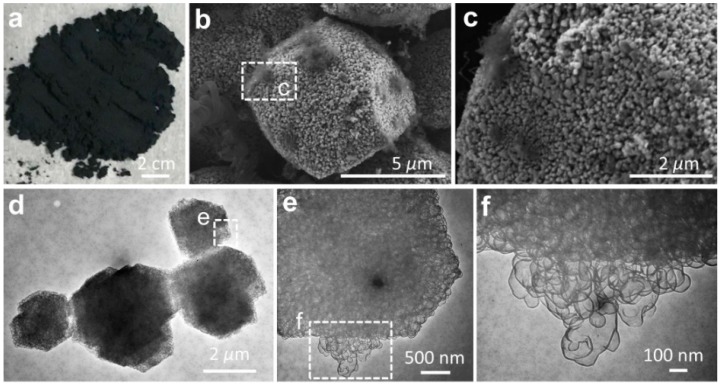
Structural features of G@MgO. (**a**) Digital photo of G@MgO powder. (**b**,**c**) SEM micrographs of individual G@MgO particles showing epitaxial growth of graphene at MgO. (**d**–**f**) TEM images of G@MgO resolving the intimate interfacial bonding between the graphene sheets and host MgO laminates.

Using the activated MgO granules as the template, the graphene sheets were synthesized by the CVD method (Figure 2a). Our hypothesis on the synthetic route to graphene at the MgO template (G@MgO) was examined by SEM and TEM observations (Figure 2b–f). At the surfaces of MgO particles, graphene entities were closely attached to the template without the trace of individual carbon sheets (Figure 2b,c). High-resolution TEM images of G@MgO shows the existence of numerous few-layer graphene sheets grown from the hexagonal template units (Figure 2d–f). It is important to note that the basic hexagonal crystalline sheets constituting the MgO templates were closely wrapped by the graphene sheets with dense mesopores [21]. The CVD-grown graphene was characterized by a sheet-like morphology resembling the initial morphology of MgO template, essentially arising from the formation of ordered carbons triggered by the crystalline entities of activated MgO particles. The role of the template was also verified by the observation of mesopores for the graphene sheets, in line with the structural features of MgO [22].

**Figure 3:**
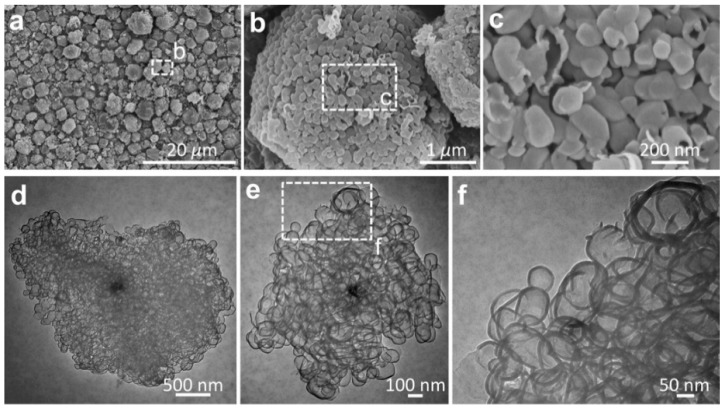
(**a**–**c**) SEM images and (**d**–**f**) TEM micrographs showing the mesopores of graphene. The host MgO templates were etched in 1 M HCl solution prior to the morphological observations.

The structural features of graphene entities were further examined by etching the host MgO particles, as shown in Figure 3. The graphene sheets were characterized by interconnected pores with a pore size centered in the range of 50–100 nm (Figure 3a–c). Figure 3d–f reveals the high-resolution observation of morphological features for the typical graphene entities, which were composed of few-layer mesopores with high transparence. The morphological observations suggest that the proceeding base growth was probably triggered by the crystalline MgO substrate, leading to the creation of nanosized graphitic cages encapsulating the underlying MgO. Wardle et al. reported the formation of turbostratic carbon nanotubes and nanofibers at the nanosized titania, yielding the presentation of a quantitative lift-off model in which several layers of carbon nanosheets lift off from the high-curvature corners of the template [23]. Here, we provide another example supporting the hypothesis in the case of the metal-oxide catalysts.

**Figure 4:**
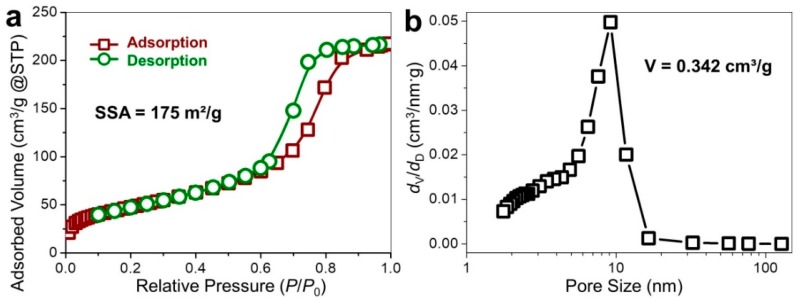
Porosimetry on graphene deposited on MgO. (**a**) N_2_ adsorption-desorption isotherms as a function of equilibrium relative pressure and (**b**) pore size distribution of graphene after etching the host MgO templates.

Measurements of surface area and porosity of the microporous graphene were conducted using N_2_ adsorption, as illustrated in Figure 4. The N_2_ adsorption-desorption isotherms (Type IV) allow us to hypothesize that the CVD grown graphene was primarily composed of mesopores (Figure 4a), giving rise to the significantly increased adsorption volume that corresponds to the filling of mesopores (in the *P*/*P*_0_ range of 0.2–0.8) [24,25]. It was accompanied by the ultimate rise (*P*/*P*_0_ > 0.8) that pointed to the capillary condensation in the finite volume of the mesopores. The specific surface area (SSA) of graphene was evaluated to be 175 m^2^/g, revealing the predominance of mesopores during the CVD growth at the MgO template. This was in line with the pore volume of 0.342 cm^3^/g, essentially arising from the existence of mesopores with probable collapsing of neighboring pores [26].

**Figure 5:**
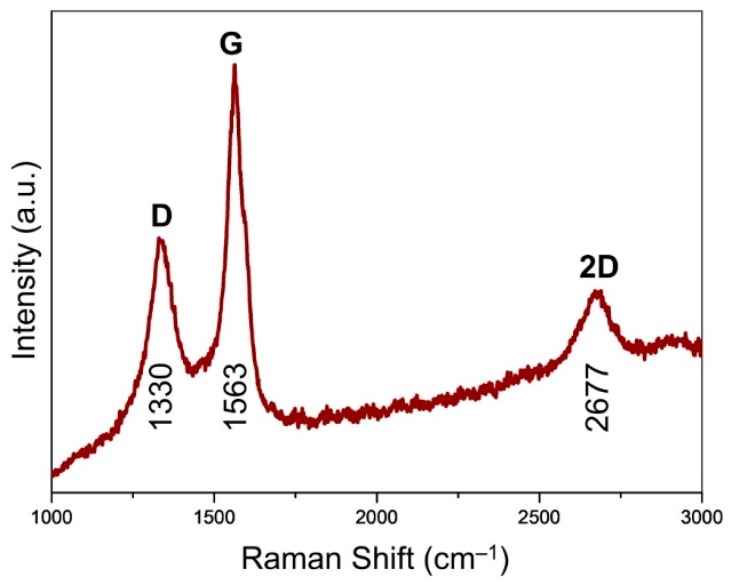
Raman spectrum of the CVD-grown graphene at the MgO template.

Raman spectroscopy affords an assessment of the ordering and graphitization degree for the carbons in the CVD-grown graphene (Figure 5). The maximum was centered at 1563 cm^−1^, assigned to the G band of graphite [27]. This indicates the existence of sp^2^ carbons, which trigger the stretching motions of E_2g_ in-plane bonds. Unlike the generally observed peak at around 1580 cm^−1^, the downshift of G band was probably due to the existence of numerous mesopores (Figure 3f), which was analogous to the case of carbon nano-onions featuring intensive curvature effect [28]. It was accompanied by the observation of D band located at 1330 cm^−1^, pointing to the existence of structural defects of graphene. The 2D band (excited by a double-resonant Raman process) located at 2677 cm^−1^ was attributed to the double resonance effects of the D band, indicating the existence of few-layer graphene presented in G@MgO [20,29]. The presence of ordered and graphitized carbons attached to the MgO particles, instead of normal graphene oxide, probably contribute to the promotion of interfacial adhesion and TC [30].

**Figure 6:**
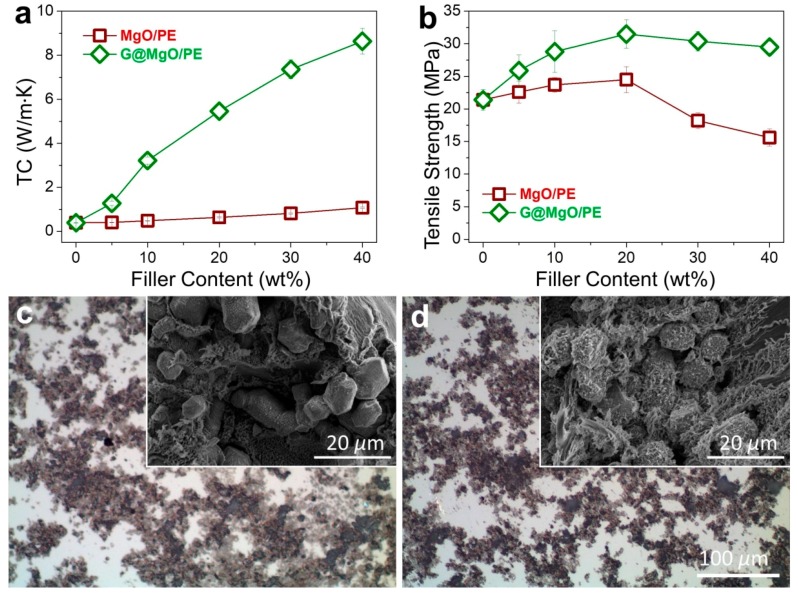
Property improvements of polymer composites with the assistance of G@MgO. Plots of (**a**) TC and (**b**) tensile strength as a function of the filler loadings. Optical images of (**c**) polyethylene/MgO (70/30) and (**d**) polyethylene/G@MgO (70/30) showing the improved dispersion of particles, the inset SEM micrographs display the representative fracture surfaces after tensile failure, respectively.

To examine the unique functionality of the hierarchical structure design, the G@MgO particles were melt-compounded with polyethylene, while the MgO-filled composite counterparts were fabricated. The presence of graphene layers is expected to bridge the polymer matrix and the host MgO entities, thus conferring the enhancements of TC and mechanical properties. The plots of TC values in Figure 6a reveal the limited increase of TC for polyethylene composites with the addition of MgO, increasing from 0.39 W/m∙K for pristine polyethylene to 1.08 W/m∙K. It is in line with the available literature reporting the use of metal and metal oxide to improve the TC of polymer composites, generally yielding the limited improvements due to the distinct thermal resistance at the interfaces. In contrast to the poor performance of MgO-filled polyethylene, marvelous increase of TC was observed for polyethylene/G@MgO composites. With the presence of 20 wt % G@MgO, the TC of polyethylene composite was increased to 5.46 W/m∙K, falling into the category of high TC. Upon the increase of filler loadings, the TC was further promoted to 8.64 W/m∙K for polyethylene/G@MgO (60/40).

In addition to remarkable enhancements in TC properties, G@MgO significantly enhanced the mechanical properties of polyethylene, as manifested by the measurements of tensile properties (Figure 6b). Although addition of MgO particles slightly pushed up the tensile strength to around 25 MPa for PE/MgO (80/20) from the initial value of 21.2 MPa for pristine polyethylene, the value fell to 15.6 MPa with the existence of 40 wt % probably due to the agglomeration of incorporated particles. Upon addition of G@MgO, the polyethylene composites witnessed large promotion of tensile strength to around 30 MPa without discernable decrease at high loadings of over 30 wt %, giving a combination of high TC and strength for the case of polyethylene/G@MgO (60/40). The optical images of the composites show the dispersion morphology for polyethylene composites, revealing the improved dispersion in polyethylene/G@MgO (70/30) compared to the counterpart (Figure 6c,d). This was mainly ascribed to the enhanced interfacial adhesion between polyethylene matrix and G@MgO, as manifested by the observation of tenacious ligaments wrapping the G@MgO particles (inset SEM image in Figure 6d). This was in contrast to the smooth surfaces of MgO particles observed for the polyethylene/MgO (70/30), indicating the poor interfacial properties that were frequently reported for the traditional inorganic particles (inset SEM image in Figure 6c). The unusual combination of high TC and mechanical robustness confers great promise in the heat sink materials (e.g., heat-conducting plates for electronics). The design principles of the hierarchical heat-conducting fillers are significant for engendering functional particles with improved surface properties. 

## 4. Conclusions

Using a precipitation method, microsized MgO granules with a controlled geometrical size were synthesized, allowing the simultaneous realization of high porosity and surface activity. By incorporation of carbon and nitrogen source, the MgO templates were ready to induce the formation of few-layer graphene nanosheets, which allowed the creation of tenacious linkages between graphene and template. The structural features allowed the suppression of intrinsically strong intersheet π-π stacking of graphene, as well as the desired exfoliation and dispersion of graphene in the polyethylene matrix. More importantly, the incorporation of G@MgO into polymer composites largely pushed up the thermal conductivity, climbing from 0.39 W/m∙K for pristine polyethylene to 8.64 W/m∙K for polyethylene/G@MgO (60/40). Meanwhile, the simultaneous promotion of mechanical properties was observed after the addition of G@MgO, reaching a tensile strength of around 30 MPa until the content of 40 wt %, in contrast to the noteworthy decline of tensile strength for MgO-filled composites with over 20 wt % fillers. The concept of pre-programmed intercalation signifies a facile method to control the morphology and regioselectivity of graphene, laying down the essential prerequisites for the fabrication of high-performance polymer composites with a combination of high thermal conductivity and mechanical properties.
